# Pyrazine analogues from wolf urine induced unlearned fear in rats

**DOI:** 10.1016/j.heliyon.2017.e00391

**Published:** 2017-08-30

**Authors:** Makoto Kashiwayanagi, Sadaharu Miyazono, Kazumi Osada

**Affiliations:** a Department of Sensory Physiology, Asahikawa Medical University, Asahikawa, Hokkaido, Japan; b Division of Physiology, Department of Oral Biology, School of Dentistry, Health Sciences University of Hokkaido, Ishikari-Tobetsu, Hokkaido, Japan

**Keywords:** Neuroscience

## Abstract

Urine excreted from the common grey wolf (*Canis lupus*) contains a kairomone, inducing fear-related behaviors in various mammals. Numerous fear-inducing substances activate neurons at the main and/or accessory olfactory bulb (AOB), medial and central amygdala, and hypothalamus. Our previous study showed that the mixture of pyrazine analogues (P-mix) contained in wolf urine induced avoidance and fear-related behaviors in laboratory mice and Hokkaido deer (*Cervus nippon yesoensis*), a species native to Japan. Exposure to wolf urine or P-mix induced expression of Fos, a marker of neuronal excitation, in the AOB of mice. In the present study, we explored the effects of P-mix on fear-related behaviors and Fos-expression in rats. Exposure to P-mix induced avoidance and immobilization in rats, while that to a mixture of *i*-amyl acetate, linalool and R(+)-limonene (O-mix), which generate floral and fruity odors, induced avoidance but not immobilization. P-mix but not O-mix increased Fos-immunoreactivity of the AOB, medial and central amygdala, and hypothalamus of rats. The present results suggest that P-mix odor induces unlearned fear-related behaviors in rats.

## 1 Introduction

Prey animals are equipped with sensory systems for predator detection. Predator odors such as cat odor, trimethyl thiazoline (TMT), which is a component of fox feces, and ferret odor induce fear-related behaviors in rats (Wallace and Rosen, 2000; Dielenberg et al., 2001; Blanchard et al., 2001; Roseboom et al., 2007). Analysis of the feces of the gray wolf shows that the diets of these animals are diverse and include smaller prey species such as rodents in addition to ungulates (Stahler et al., 2006). This suggests that rodents are afraid of wolves. In fact, exposure to the urine of wolves induces avoidance and the flat-back approach in rats (Fendt, 2006). Previously, we identified pyrazine derivatives in wolf urine that induced fear-related responses in mice and Hokkaido deer (*Cervus nippon yesoensis*), a species native to Japan (Osada et al., 2013; 2014; 2015).

Many mammalian species have two major olfactory systems: a main olfactory and a vomeronasal system. The olfactory sensory neurons (OSN) respond to various general odorants including kairomones, which provoke an aversive effect in interspecies animals. Sprague-Dawley rats and mice avoid 2-phenylethylamine (2-PEA), which is contained in excretions of mountain lions, bobcats and wolves (Ferrero et al., 2011). 2-PEA activates mouse OSNs via olfactory trace amine-associated receptor 4, which is expressed at the main olfactory epithelium but not at the vomeronasal sensory epithelium. In addition, unlearned avoidance responses to 2-PEA were maintained in mice lacking TrpC2, which is a key transduction channel in the vomeronasal sensory neurons (VSN) (Ferrero et al., 2011), indicating that 2-PEA induces avoidance via the main olfactory pathway.

The main olfactory bulb (MOB) and accessory olfactory bulb (AOB) are the primary brain regions involved in the major olfactory transmitting pathway in rodents. Cat odor but not TMT caused pronounced activation of Fos, a marker of neuronal excitation, in the AOB, a primary brain region that receives information of pheromones and kairomones from the VSNs, of rats, suggesting that cat odor induces fear-related responses via the vomeronasal system (McGregor et al., 2004; Staples et al., 2008a; b). Wolf urine or a mixture of three pyrazine derivatives (P-mix) induced an increase in Fos-immunoreactive (Fos-ir) cells in the AOB in mice (Osada et al., 2013; 2015). The amygdala is an important brain region for threat detection and the elicitation of fear-related behavior (Asok et al., 2013). The medial amygdala (MeA) receives direct projections from the AOB (Scalia and Winans, 1975) and indirect projections from the MOB (Sah et al., 2003). Cat odor, TMT, and ferret odor induce excitation of neurons at the central amygdala (CeA) and MeA of rats (Day et al., 2004; Campeau et al., 2008; Sharma et al., 2014). The accessory olfactory component of the amygdala projects to the medial hypothalamus (Swanson and Petrovich, 1998). Information of predator cues conducts to the predator-responsive circuit composed with the ventromedial hypothalamus, dorsal medial part (VMHdm), anterior hypothalamic nucleus (AHN) and dorsal premammillary nucleus (PMD) at the hypothalamus (Gross and Canteras, 2012).

In the present study, we first asked the question whether P-mix would induce fear-related responses in rats. It was expected that P-mix would induce excitation of neurons at the fear-related brain regions such as amygdala and hypothalamus in addition to the AOB of rats. Then, we examined the Fos-immunoreactive structure at the AOB, MeA, medial division of central amygdaloid nucleus (CeM), and hypothalamus after exposure to P-mix to explore whether P-mix induces neuronal excitation in these brain regions of the rats.

## 2 Materials and methods

### 2.1 Animals

All experiments were carried out in accordance with the Guidelines for the Use of Laboratory Animals of the Asahikawa Medical University and approved by the Committee of Asahikawa Medical University for Laboratory Animal Care and Use (approval ID: 13009). A total of 32 female Donryu rats (11–14 weeks old) were used. Donryu strain rats were derived from albino rats and established in Japan. The rats were obtained from Sankyo Laboratory Co. (Sapporo, Japan). Rats were kept in a room maintained at 22 °C with a photoperiod of 12 h: 12 h (non-reversed 12 h light/dark cycle). Two or three animals were housed per cage. All rats had free access to food and water. More than 2 weeks were allowed to the environment after the arrival from the company. Rats were experimentally innaïve animals and were not acclimatized for the experimental procedures. All experiments were conducted during light phase.

### 2.2 Preparation of odor cocktails

The P-mix was composed of equal volumes of 2,6-dimethylpyrazine, 2,3-diethyl-5-methylpyrazine and 2,3,5-trimethylpyrazine (Tokyo Chemical Industry Co., Ltd., Tokyo, Japan). In the present study, 2,3-diethyl-5-methylpyrazine was used as a P-mix constituent instead of 3-ethyl-2,5-dimethyl pyrazine, which was previously included in a pyrazine cocktail to induce avoidance and fear-related behaviors in mice and deer (Osada et al., 2013; 2014). A large amount of 2,3-diethyl-5-methylpyrazine was contained in wolf urine (#21 in Table 1; Osada et al., 2013). Exposure to 2,3-diethyl-5-methylpyrazine alone induces freezing in mice (Osada et al., 2017). The time spent immobilized by mice after exposure to P-mix was similar to that to the previous one (data not shown). The O-mix was composed of equal volumes of linalool, R(+)-limonene and *i*-amyl acetate (Wako, Japan). These odorants have been used in studies of the rat olfactory system (Xu et al., 2000; Briand et al., 2000; Clarin et al., 2010).

### 2.3 Avoidance

**Fig. 1:**
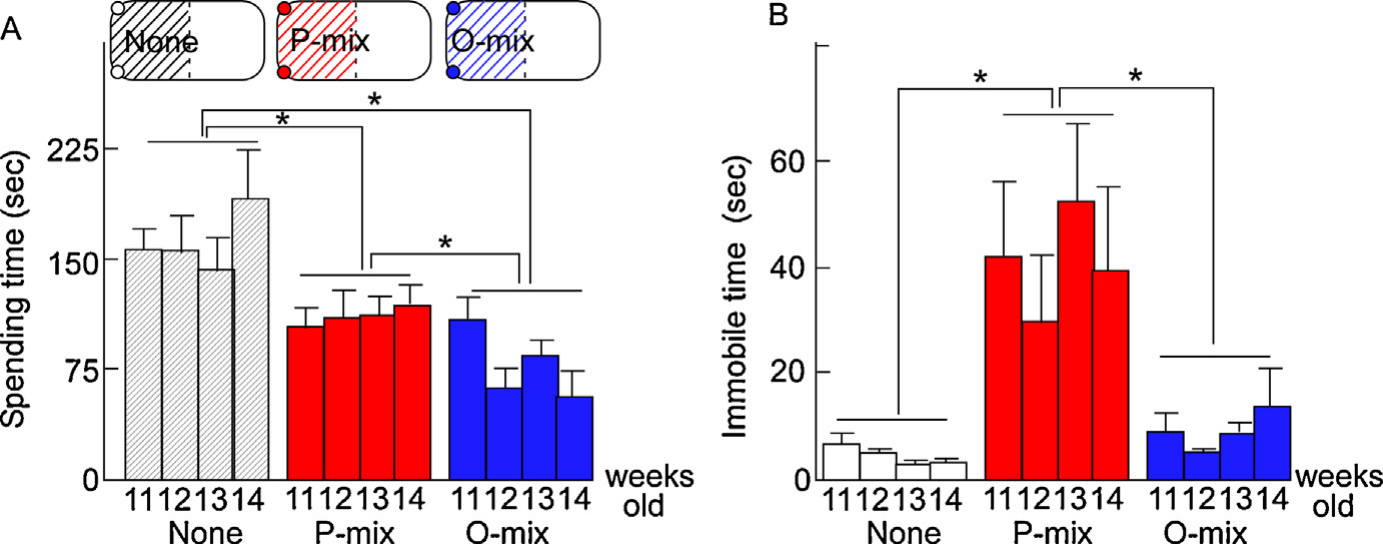
Avoidance and immobilization induced by P-mix or O-mix in rats. A schematic of the experimental arena and time spent in the odorized zone (A). (A) Time spent in the cage halves containing petri dishes without odor mixture (white), or with P-mix (red) or O-mix (blue) in the two corners over 5 min. (B) Time spent immobilized over a 2-min period beginning at 3–5 min after exposure to P-mix (red), O-mix (blue), or no odorants (white). n: 6 (control), 6 (P-mix), 7 (O-mix). Values are the mean ± SE of data. *p < 0.05.

Two plastic dishes (diameter: 4 cm), each containing a piece of filter paper with 100 μl of P-mix, O-mix or no odorants, were placed in two of the four corners of the cage (size: W260 x D425 x H200 mm) and covered with the animal bedding made of paper to explore effects on avoidance (schematic in Fig. 1A). An animal's movements were recorded by a video camera. We measured the amount of time spent by individual rats in the odor-bearing (red shadow or blue shadow) half and odor-less (black shadow) half of the cage. During 5 min after exposure to P-mix, O-mix of none odor, the length of time each nose of rats entered in half zone (shadowed area in Fig. 1A) was measured.

### 2.4 Immobilization

P-mix (200 μl) or O-mix (200 μl), which was dissolved in 20 ml Milli-Q water, was applied to the animal bedding in the cage (size: W260 x D425 x H200 mm). An animal's movements were recorded by a video camera. Time spent in immobilization was measured during a total absence of body or head movement except that associated with breathing from 3 to 5 min after exposure to odorants.

### 2.5 Tissue processing and immunohistochemistry

The animals were deeply anesthetized with ether and pentobarbital sodium (35 mg/kg) 90 min after exposure to P-mix, O-mix or no odorant. P-mix (200 μl) or O-mix (200 μl) was applied to the animal bedding in the cage (size: W260 x D425 x H200 mm). The animals were then perfused through the heart with phosphate-buffered saline (PBS), followed by fixation with 4% paraformaldehyde. The brains were removed, and the tissue samples were soaked in identical fixative solution overnight, then sliced in a serial manner on a vibratome at a thickness of 100 μm. The free-floating sagittal sections were first treated with 0.6% H_2_O_2_ for 15 min in PBS with 0.4% Triton X–100 (PBSx), followed by two washes with PBSx. After 1-hr incubation in 3% normal goat serum, the sections were incubated overnight at room temperature with anti-c-Fos polyclonal antibody (1:16000, Ab-5; Merck Millipore, Billerica, MA) in PBSx. The sections treated in this manner were then rinsed in PBSx and incubated with biotinylated goat anti-rabbit IgG (1:4000; Vector, Burlingame, CA) for 1 h. The sections were rinsed again in PBSx, incubated with ABC (ABC Elite kit; Vector) for 1 h, and developed with DAB/H2O2 (0.05% DAB and 0.003% H_2_O_2_ in 0.05 M Tris-HCl buffer) for 12 min. After a final rinse with water, the sections were mounted, dehydrated and covered with cover slips. The thickness of dehydrated slices was about 20 μm (Utsugi et al., 2014).

Images of Fos-ir cells were photographed by a CCD camera (DP72; Olympus, Tokyo, Japan) attached to an inverted microscope (BX51; Olympus), and counted by the naked eye. The magnification immediately in front of the CCD camera was x 5. All Fos-ir cells in dehydrated sections were visualized in the same or nearly the same focal plane in our experimental equipment (Utsugi et al., 2014). The areas of the brain regions were measured using SigmaScan Pro software (SPSS Inc., Chicago, IL). The average density of individual rats was calculated from summation of the number of Fos-ir cells in 6 slices of AOBs and 2 slices of each amygdala or hypothalamus. For calculate the density of Fos-ir cells (number/mm^3^), a thickness of 100 μm was used. The edge of the border of the rostral and caudal regions of the sagittal section was identified by the characteristic shape of the AOB. The border of each region of the amygdala and hypothalamus was distinguished by overlaying a brain atlas drawing adapted from the stereotaxic atlas of the rat brain (Paxinos and Watson, 2007) on our photographs.

### 2.6 Statistical analysis

The data were analyzed using StatView for Windows (SAS Institute Inc., Cary, NC, USA). Differences in the density of Fos-ir cells at various regions of the brain, and times spent in the different regions or immobilized were analyzed using analysis of variance (ANOVA) with Tukey-Kramer's post-hoc test. Significance was established at p < 0.05. Data are given as the mean ± standard error of the mean.

## 3 Results

### 3.1 Avoidance induced by wolf urine, P-mix or O-mix in rats

First, we examined avoidance of rats in response to P-mix (Fig. 1A). We also examined the response of rats to O-mix odor, which contains more familiar odors of flowers and fruit but no wolf-urine odorants, to explore the hypothesis that rats avoid P-mix odor because it is unfamiliar to them. In the present study, the estrous cycle was not determined. Time spent in the P-mix, O-mix or odor-free half of the cages during the initial 5 min after exposure was measured once every one week between 11th and 14th week old. Repeated measures ANOVA revealed a significant group effect (F(2, 66) = 19.239, p < 0.0001). Tukey-Kramer's post-hoc testing indicated that time spent in the P-mix or O-mix half was significantly shorter than that spent in the odor-free half of the cage. It also indicated that time spent in the O-mix half was significantly shorter than that spent in the P-mix half of the cage. These results suggested that rats avoid odors of both P-mix and O-mix.

### 3.2 P-mix-induced immobilization in rats

To explore whether odors of P-mix or O-mix induce fear-related behavior, the time spent immobilized by rats after exposure to P-mix or O-mix was measured (Fig. 1B). Repeated measures ANOVA revealed a significant group effect (F(2, 73) = 22.914, p < 0.0001). Tukey-Kramer's post-hoc testing indicated that P-mix induced a longer immobilization of rats than did O-mix or no odor. These result suggested that P-mix odor but not O-mix odor induced immobilization.

### 3.3 Fos-immunoreactivity in the AOB of rats after exposure to P-mix and O-mix

**Fig. 2:**
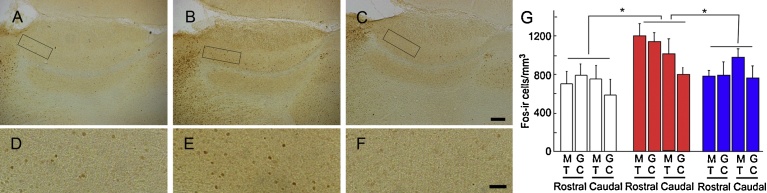
Fos-immunoreactivities at the AOB of rats after exposure to P-mix or O-mix. Sagittal sections of the AOB after exposure to no odorants (A and D), P-mix (B and E), and O-mix (C and F). The frame in the mitral cell layer in the rostral AOB (A, B and C) indicates the area shown in D, E and F, respectively. Scale bar: 200 μm (C), 50 μm (F). The densities of Fos-ir cells (number of cells/mm^3^) in the mitral cell layer (MT) and granule cell layer (GC) at the rostral and caudal regions of the AOB after exposure to no odorants (white), P-mix (red) and O-mix (blue) are also shown. Density was analyzed using a one-factor ANOVA with Tukey-Kramer's post-hoc test. n: 4 (control), 4 (P-mix), 5 (O-mix). Values are the mean ± SE. *p < 0.05.

To explore whether P-mix also induces excitation of neurons in the AOB of rats, we investigated the Fos-ir structure after exposure to P-mix. Exposure to P-mix resulted in a large amount of Fos-expression in the mitral and granule cell layer at the rostral AOB (Fig. 2B and E). In contrast, exposure to no odorants or O-mix did not induce remarkable Fos-immunoreactivity in the AOB (Fig. 2A, C, D and F). These results suggested that P-mix but not O-mix induced excitation of the vomeronasal system of the rats.

Fig. 2G shows the density of Fos-ir cells (number/mm^3^) in serial sagittal sections of the AOB of rats after exposure to P-mix or O-mix. The data from each group were cast into a three-factor ANOVA with stimulation, regions (rostral and caudal) and layers (mitral cell and granule cell layers) as factors. This analysis revealed a main effect of stimulation (F(2, 39) = 7.427, p = 0.0018). Tukey-Kramer's post-hoc testing indicated that the density of Fos-ir cells in rats after exposure to P-mix was higher than that in those exposed to no odorant cocktail or those exposed to O-mix, while the density of Fos-ir cells after exposure to O-mix was similar to that after exposure to no odorant cocktail.

### 3.4 Fos-immunoreactivity in the amygdala and hypothalamus of rats after exposure to P-mix and O-mix

**Fig. 3:**
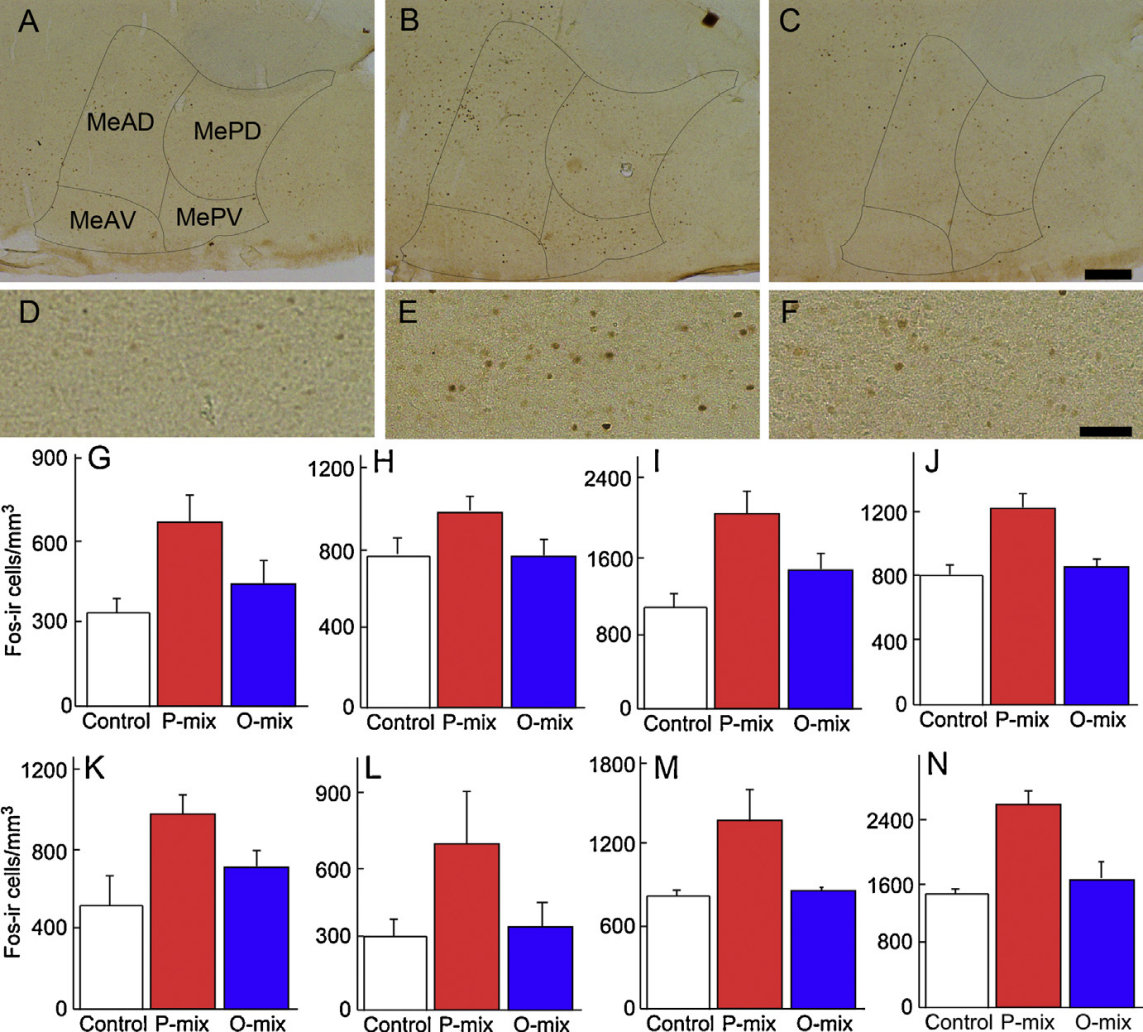
Fos-immunoreactivities at the amygdaloid nucleus and ventromedial hypothalamic nucleus of rats after exposure to P-mix or O-mix. Sagittal sections at the amygdaloid nucleus (A, B and C) and enlarged sections at the MePV (D, E and F) after exposure to no odorants (A and D), P-mix (B and E), or O-mix (C and F). Scale bar: 200 μm (C), 50 μm (F). Density of Fos-ir cells (number of cells/mm^3^) after exposure to no odorants, P-mix or O-mix in the MePV (G), MePD (H), MeAV (I), MeAD (J), CeM (K), VMHdm (L), AHN (M) and PMD (N). n: 4 (control), 4 (P-mix), 5 (O-mix). Values are the mean ± SE.

Information concerning predator odors is transmitted to the MeA, CeA, and hypothalamus (Roseboom et al., 2007; Staples et al., 2008a; b; Perez-Gomez et al., 2015). We investigated the Fos-immunoreactive structure at the amygdaloid nucleus and hypothalamus of rats after exposure to P-mix or O-mix (Fig. 3). Exposure to P-mix resulted in a large amount of Fos-expression in the MeAD, MeAV and MePV (Fig. 3B and E). In contrast, exposure to O-mix did not induce remarkable Fos-immunoreactivity in the medial amygdala (Fig. 3C and F).

Fig. 3G-N shows the density of Fos-ir cells (number/mm^3^) in sagittal sections of the amygdala nucleus and hypothalamus of rats after exposure to P-mix or O-mix. Analysis of the data at the medial amygdala (Fig. 3G-J) of each group with a two-factor ANOVA (factors: stimulation and region) revealed main effects of stimulation (F(2, 40) = 14.271, p < 0.0001) and regions (F(3, 40) = 38.009, p < 0.0001), and an interaction (F(6, 40) = 2.474, p = 0.0395). Tukey-Kramer's post-hoc testing indicated that the density of Fos-ir cells at the MeA after exposure to P-mix was higher than that without P-mix or after exposure to O-mix. These results indicate that P-mix induces excitation of neurons at the medial amygdala of rats.

A one-factor ANOVA analysis of the density of Fos-ir cells at the CeM (Fig. 3K) revealed a main effect of stimulation (F(2, 10) = 4.109, p = 0.0498). Tukey-Kramer's post-hoc testing indicated that the density of Fos-ir cells at the CeA in rats after exposure to P-mix was higher than that without such exposure.

A two-factor ANOVA analysis of the density of Fos-ir cells at the hypothalamus (Fig. 3L-N) also revealed main effects of stimulation (F(2, 29) = 17.705, p < 0.0001) and region (F(2, 29) = 69.294, P < 0.0001). The densities of Fos-ir cells at these regions with P-mix exposure were higher than without P-mix exposure and with O-mix exposure (Tukey-Kramer's post-hoc test). These results indicate that P-mix but not O-mix induces excitation of neurons at these regions of the hypothalamus of rats.

## 4 Discussion

In rats, urinary pheromones induce more pronounced Fos-expression at the granule cells, which are interneurons, than that at mitral cells, which innervate to a higher level of olfactory brain regions (Inamura et al., 1999; Honda et al., 2008). In the pheromonal reception involved in reproduction, modification of pheromonal information generated by interneurons is very important for individuals to learn, and changes in the neural connections have been shown to occur between the mitral cells and granule cells at the AOB in order to form memories to preserve own species (Kaba et al., 1994; Matsuoka et al., 1997). In contrast, cat odor (McGregor et al., 2004; Staples et al., 2008a; b) induced similar levels of Fos-expression in the mitral and granule cells of the rat AOBs. After exposure to P-mix, level of Fos expression in the mitral cells was also similar to that in the granule cells. These results suggest that it is important to transmit unlearned fear information without unnecessary modification by interneurons at the AOB in order for individuals to defend themselves from danger.

TMT exposure induced Fos expression in many brain regions, particularly in the MePV (Jantizky et al., 2015). Strong initial fear-related behaviors to cat odor were accompanied by the expression of c-Fos ir-cells at the MePV (Staples et al., 2008b). The MeAV is considered to play a role in orienting responses to chemosensory cues and fear-related behaviors elicited by the odor of predators (Novaes and Shammah-Lagnado, 2011). Axons from the rostral and caudal regions of the AOB terminate in the MeAV (Mohedano-Moriano et al., 2007). The major target of the MeAV is the ventromedial hypothalamic nucleus (Novaes and Shammah-Lagnado, 2011). Neurotoxic lesions of the medial amygdala of rats produce decrements in fear-related behaviors to cat odors (Blanchard et al., 2005). Similarly, inactivation of the medial amygdala with infusion of muscimol, a GABA_A_ receptor agonist, blocks TMT-induced freezing of rats (Muller and Fendt, 2006). As shown in the present study, P-mix induced an increase in Fos-immunoreactivity at the MePV and MeAV. The predator-responsive circuit composed with the VMHdm, AHN and PMD at the hypothalamus receives information of predator cue (Gross and Canteras, 2012). Avoidance and freezing induced by exposure of rats to a cat are eliminated by cell body-specific chemical lesions by ibotenic acid at the PMD (Canteras et al., 1997). Exposure of rats to P-mix increased Fos-ir cells in these regions of the hypothalamus (Fig. 3). These results suggested that the medial amygdala and hypothalamus are involved in the fear-related behavior of rats induced by P-mix.

Cues that are associated with painful stimuli (Gross and Canteras, 2012) as well as ferret odor, TMT and P-mix have all been shown to activate the CeA (Roseboom et al., 2007; Sharma et al., 2014). In the present study, P-mix induced Fos-ir cells at the CeM while O-mix did not. Infusions of the AMPA receptor antagonist 2, 3-dihydroxy-6-nitro-7-sulphamoylbenzo(F)-quinoxaline into the CeA blocked fear-potentiated startle, indicating that the CeA is involved in conditioned responses to fear stimuli (Walker and Davis, 1997). Temporary inactivation or lesioning of the CeA did not block TMT- or cat odor-induced freezing in rats, suggesting that the CeA is not critically involved in the induction of unlearned fear by TMT or cat odor (Fendt et al., 2003; Li et al., 2004). Therefore, it is possible that the CeA does not play a predominant role in the induction of unlearned fear by P-mix, even though P-mix activates neurons at the CeA.

Deer respond aversively to the odor of urine excreted from three other predators: wolves, bobcats and coyotes (Sullivan et al., 1985; Swihart et al., 1991). Interestingly, P-mix also induces fear-related behaviors in Hokkaido deer (Osada et al., 2014). In Japan, wolves were feared and hunted, and they had disappeared from the country by 1905 (Walker, 2005). Therefore, recent generations of Hokkaido deer have never encountered wolves. Similarly, P-mix has been shown to evoke immobilization in laboratory mice (Osada et al., 2013). In the present study, P-mix but not O-mix induced immobilization and expression of Fos-ir cells at the brain area related to fear information, such as MeA and hypothalamus. These results provide support for the hypothesis that P-mix odor induces unlearned fear in these animals.

## 5 Conclusion

Rats avoided both the P-mix and O-mix odors, but displayed immobilization only in response to P-mix. Exposure to P-mix but not O-mix induced expression of Fos-immunoreactivities at the AOB, amygdala ad hypothalamus of rats. The present results indicated that P-mix, pyrazines involved in wolf urine, induced unlearned fear in rats.

## Declarations

### Author contribution statement

Makoto Kashiwayanagi: Conceived and designed the experiments; Performed the experiments; Analyzed and interpreted the data; Contributed reagents, materials, analysis tools or data; Wrote the paper.

Sadaharu Miyazono, Kazumi Osada: Wrote the paper.

### Funding statement

This work was supported by Asahikawa Medical University, Japan.

### Competing interest statement

The authors declare no conflict of interest.

### Additional information

No additional information is available for this paper.
